# Supplementary material for: Are carbon emissions trading and green financial instruments synergistic? -Comprehensive quantitative research based on content analysis
Source: PLoS One. 2024 Mar 7;19(3):e0298601. doi: 10.1371/journal.pone.0298601 (PMC10919601; doi:10.1371/journal.pone.0298601)
Supplement: S1 Data — (ZIP) [file pone.0298601.s001.zip › Data/地级市Z_F工作报告.zip!/╔┬╬≈/═¡┤¿╩╨/└┤╫╘╣1⁄2╓┌║┼í╛┬φ┐╦╩2╛▌═°í┐_2013.docx]

各位代表：

　　我代表市人民政府向大会报告工作，请予审议。请政协委员和各位列席人员提出意见。

　　一、去年工作回顾

　　2012年，面对宏观形势的严峻挑战，在省委、省政府和市委的坚强领导下，全市人民以邓小平理论、“三个代表”重要思想、科学发展观为指导，紧扣提速发展、加快转型、富民强市主线，实施工业强市、文化兴市、生态立市三大战略，攻坚克难，开拓进取，完成了市十五届人大二次会议确定的目标任务。

　　全年实现生产总值282.92亿元，增长15.8%；固定资产投资201.81亿元，增长38.3%；社会消费品零售总额62.46亿元，增长17.3%，这三项指标增速均居全省第一。全市财政总收入41.59亿元，同口径增长17.3%；地方财政收入21亿元，同口径增长31.3%， 增速居全省第三；城镇居民人均可支配收入21929元，增长16.8%，增速居全省第二；农民人均纯收入7134元，增长17.4%，增速居全省第五；万元GDP能耗下降3.62%，降幅居全省第三；四项主要污染物减排任务超额完成，降幅居全省前列。

　　（一）工业在困境中持续增长。煤炭、水泥、铝业、电力等支柱产业负重前行，增加值增长21.9%。装备制造、食品、医药等接续产业发展势头较好。29种主要工业产品中24种产量保持增长，原煤增长26.3%，水泥增长40.1%。工业产销率98%。规模以上工业增加值157.69亿元，增长21.6%。

　　（二）重点项目建设取得突破。实施重点项目115个，完成投资130.65亿元，21个项目建成投运，32个项目开工。“科学发展双十行动”完成投资65.33亿元。董家河产业园被列为全省循环经济试点单位和产业示范基地。国家发改委同意华能铜川电厂二期项目开展前期工作。

　　（三）现代农业发展较快。全年财政支农资金8.9亿元，增长29.6%。新增省级现代农业园区6个。伊利耀州万头奶牛养殖项目开工。新栽鲜干果经济林13.09万亩，苹果总产64.7万吨。大樱桃生产技术推广成果获省上一等奖。粮食总产24.4万吨，实现“九连丰”。农林牧渔业增加值19.47亿元，增长6.3%，增速居全省第二。

　　（四）休闲养生产业发展势头良好。大唐养生园完成投资3.05亿元，主要工程全面铺开，生态湖等项目完工。照金红色旅游名镇完成投资2.83亿元，照金纪念馆、群众安置小区等7个项目主体完工。联合设立了2.5亿元生物医药创投基金。举办了第二届孙思邈中医药文化节。实现旅游综合收入30.8亿元，增长61%。文化产业增加值4.53亿元，增长34.4%，增速居全省第二。

　　（五）改革开放取得进展。完成工商、质监行政管理体制调整工作。市本级财政支出全部集中支付。发行企业债券8亿元。社会融资规模187.22亿元，增长53.5%。非公经济总产值增长22%。外贸进出口总额1241.6万美元，增长22.1%。招商引资到位资金114.8亿元。

　　（六）城乡建设统筹推进。编制了全市城乡一体化建设等规划。城乡基础设施投资13.2亿元。拆除危旧建筑26万平方米。城市空气质量二级以上天数329天。国家卫生城市创建扎实推进，全国绿化模范单位创建已申请全国绿化委评定。黄堡示范镇建设完成投资4.26亿元。农村移民搬迁6658人，解决了3.05万农村人口饮水安全问题，搬迁地质灾害隐患点群众2319人，农村居民进城落户1.76万人。

　　（七）民生工作力度加大。投资28.87亿元，实施民生项目584个，建成406个。保障房竣工2.13万套，新增租赁补贴户3010户，1.01万户困难群众喜迁新居，锦绣新城建设受到省委、省政府表扬。全年发放小额担保贷款3.5亿元，新增城镇就业1.8万人，城镇登记失业率3.5%。落实社会救助资金2.25亿元，城乡低保10.23万人。城镇职工医疗保险住院报销比例85.3%，新农合报销比例81.3%，均处于全省领先水平。为5.3万高龄老人发放生活保健补贴。在全省率先实现了安全校车全覆盖，建成公办幼儿园26所。图书馆服务一体化国家示范项目顺利推进。新建改造农贸市场4个、标准化菜市场5个，新增82辆放心早餐销售车和10辆蔬菜直销车。居民消费价格涨幅回落到2.8%。

　　（八）社会管理创新不断提升。红旗社区“3+3+X”管理模式在全省推广。人口自然增长率3.62‰。安全生产被评为全省先进单位。实现了信访维稳“一控制、两下降”目标，信访考核全省排名第一。社会治安满意率92.4%，被评为全省社会治安综合治理优秀市。

　　（九）政府自身建设有所加强。办理人大代表建议35件、政协提案275件，办复率100%。政务公开不断扩大，政府门户网站浏览量超过700万人次。强化行政监察、审计监督，廉政勤政建设进一步加强。

　　各位代表，这些成绩来之不易，是市委统揽全局、科学决策的结果，是市人大、市政协和社会各界支持监督的结果，更是全市人民团结拼搏、真抓实干的结果。在此，我代表市人民政府，向全市人民，向关心支持铜川发展的社会各界人士，表示崇高的敬意和衷心的感谢！

　　同时，我们也清醒地看到，在前进道路上还有诸多困难和问题，政府工作中还存在不少缺点和不足。经济对资源的依赖度依然较高，非资源性接续产业发展不足，县域经济实力不强，创新创业活力不足；大项目、高新技术企业少，经济增长内生动力不够强；能耗水平依然偏高，一些行业和区域的环境污染问题突出；城市规划建设滞后，管理机制和综合配套功能不健全，整治投资环境和市容环境力度还需加大；安全生产基础不牢，促进群众增收、创新社会管理、改善民生的任务繁重；一些政府工作人员责任心不强、工作效率不高已经成为比较突出的问题，不主动作为和相互扯皮、相互推诿的情况时有发生，形式主义、做表面文章的现象仍然存在，直接导致一些政府工作落实不力、服务不到位。对这些困难和问题，我们必须高度重视，不掩饰、不回避，认真加以解决。

　　二、今年的主要任务

　　今年是贯彻落实党的十八大精神的开局之年，是实施“十二五”规划承前启后的关键之年，也是铜川推进提速转型发展的重要一年。当前，铜川发展机遇汇集、前景看好，加快发展的天时、地利、人和俱在，基础、环境、条件并存。我们的任务就是明晰思路鼓干劲，一以贯之抓落实，持之以恒干到底，努力推动铜川经济社会更好更快发展。

　　根据市委十一届二次全会安排，2013年政府工作的总体要求是：以党的十八大精神为指导，全面贯彻落实中央和省委省政府的决策部署，以转型发展为统揽，坚定不移地实施工业强市、文化兴市、生态立市三大战略，把保增长放在首位，加快传统产业改造提升和接续产业培育发展，切实提高经济增长质量和效益，加大城乡统筹力度，深化改革开放，大力保障和改善民生，全力推进经济强、文化兴、生态美的全国知名休闲养生城市建设。

　　主要预期目标是：生产总值增长14.5%；地方财政收入同口径增长21%；城镇、农村居民人均可支配收入分别增长16%和17%；物价涨幅控制在4%以内，城镇登记失业率控制在4.5%以内，人口自然增长率控制在5.5‰以内；万元GDP能耗下降3.4%，化学需氧量、氨氮、二氧化硫和氮氧化物排放量分别削减2.2%、2.5%、3%和5%。

　　（一）突出抓好项目建设，夯实提速转型发展的基础

　　抓项目就是抓发展。集中力量、重点突破一批承载转型、支撑转型、推动转型的重大项目。全市固定资产投资完成259亿元，增长28%。

　　加快重点项目建设。模块化推进转型发展十大项目建设，确保重点项目完成投资143亿元以上。加快华能铜川电厂二期、美鑫循环经济、伊利耀州万头奶牛养殖、照金文化旅游等项目建设。做好油气、风电、油页岩等资源勘查开发，力争年内形成10万吨原油生产能力。延西高速新区至何家坊与包茂高速连接线、耀旬公路与薛家寨连接线、耀柳路九里坡改建等工程建成通车，开工建设210国道川口至耀州段改建工程，做好合凤高速新区至照金段、西包城际高铁客运专线铜川段项目前期工作。加快龙潭水库建设，积极参与东庄水库调水的前期工作，为转型发展提供充裕的水源保障。

　　强力推进招商引资。坚持招大选优引强，充分发挥自身优势，深入挖掘特色资源，以最大的诚意、最优的服务、最好的项目，对接战略投资者。精心策划包装开展好招商活动，加大签约项目跟踪落实，促进重大项目落地，切实提高合同履约率、资金到位率和项目开工率，确保全年合同利用市域外资金200亿元以上，实际利用市域外资金80亿元以上。

　　营造良好发展环境。扎实开展“发展环境优化年”活动。继续对重点项目建设实施“三位一体”包联责任制。加强项目前期论证工作，建立项目风险预警和退出机制，坚决不上污染环境、浪费资源的项目。清理闲置土地，加强土地储备，加快项目报批，确保项目引得来、落得住、能发展。优化金融生态环境。对重点项目建设继续实行定期通报、季度考核、半年奖惩。

　　（二）加快产业转型升级，构建现代产业体系

　　产业转型升级是资源型城市转型的核心。立足铜川资源优势和产业结构特点，强化创新驱动，改造提升传统产业，积极培育接续产业，大力发展现代服务业，减少对资源的依赖度，以转型促发展，以发展促转型，实现有质量有效益可持续发展。

　　做强做大工业经济。工业是铜川的经济支柱。依托资源、彰显优势、延伸产业链、提高附加值，加快传统产业改造提升。整合煤炭资源，关闭不具备安全生产条件的矿井，推进煤电一体、煤化同步，提高煤炭资源就地转化率。聚集水泥优势产能，改造提升现有企业，建成声威三期水泥生产线，大力发展特种水泥及新型水泥制品，做到清洁生产。加快30万吨碳素、铭帝铝业等深加工项目建设，延伸铝业产业链。陶瓷产业要在继承中创新，与市场和国际接轨，加强新产品研发，加快现代陶瓷研发生产基地等项目建设，提高对经济的贡献率。突出特色、集群发展、壮大规模、打响品牌，培育发展装备制造、食品、医药等接续产业。抓好8个工业园区建设，加大基础设施建设力度，改善功能区投资环境，吸引高新企业、高端人才向园区聚集，力争每个园区落户3－5个有规模的产业项目。加强工业运行调度，强化企业管理，帮助企业增产扩销提效。全市新增规模以上企业20户以上，规模以上工业增加值增长19%。

　　发展休闲养生经济。加强与大企业、大集团合作，推进大唐养生园、中医药产业园和药王山、照金香山、玉华宫、陈炉古镇景区开发建设。加快孙思邈医德传承基地、中医药研究院建设，推进中医药大学项目，加强中医药产品及方剂研发。开发特色旅游产品，大力发展养生休闲游、红色足迹游、陶瓷文化游、生态体验游，积极发展乡村旅游，打响旅游品牌。全年实现旅游综合收入38亿元以上。培育骨干文化企业，加快文化产业发展。认真落实促进消费的政策措施，扩大网络消费和信用消费，培育小吃聚集区和养生餐饮特色品牌，搞好农超对接、放心早餐、“双百”市场、蔬菜进社区等工程建设。加强市场监管，提高产品和服务质量。全社会消费品零售总额72.75亿元，增长16.5%。

　　大力发展现代农业。夯实现代农业园区建设的责任，充分调动市、区县两个积极性，引进高新技术项目和战略投资者，加快推进7个省级、11个市级现代农业园区建设，增强园区的辐射带动能力。发展特色高效农业，加大鲜干果经济林建设，让群众在绿化中受益，在栽树中得实惠。加快西北农林科技大学铜川果树试验示范站建设，大力推广果业标准化栽培技术，加强储藏和冷链运输能力建设，打响铜川果业品牌。建设万亩现代果业示范园3个，新建标准化果园2万亩。建成万头生猪生产示范基地2个。新建百亩设施蔬菜示范基地2个。抓好粮食高产创建，确保粮食总产稳定在20万吨以上。加强气象灾害监测预警和防灾减灾体系建设。建立区县畜牧生产安全监管信息系统，改造提升20个乡镇农产品质检站。做好宜君国家平安农机示范县创建工作。抓好龙头企业培育、农民专业合作社建设和职业农民培训，鼓励和扶持家庭农场和经营大户，提高农业组织化程度。

　　（三）完善服务功能，提升城市品质

　　城市让生活更美好。创新城市发展理念，坚持大手笔规划、高标准建设、精细化管理，打造清洁铜川、绿色铜川、品质铜川。

　　坚持规划为纲。进一步完善城市总体规划，扩容发展空间。高标准编制专项规划，做好中心城区总体城市设计，打造城市特色，提升城市形象。建立铜川市地理信息数据库，建设城市规划展览馆。加强规划管理和执法监察，加大违章违规建设查处力度。

　　加快城市建设。完善市政设施，健全服务体系，改善人居环境。建立投融资平台，增强政府筹措城建资金的能力。加快新区华原西道等道路建设，完成阳光广场二期工程，实施正阳路、朝阳路架空线落地工程。抓好北市区沉陷区滑塌区治理和光明巷道路改造等工程建设。启动工矿区、铁路沿线等棚户区改造。加强城市饮用水源地保护，实施黄堡净水厂供水提质改造工程。积极引进知名餐饮商贸企业，加快铜川大厦等星级酒店及道上太阳城、正阳商业综合体等项目建设。启动城市集中供热工程。完成新区医院二期和市中医医院新区项目主体工程。坚持政府主导、市场运作，加快推进城中村改造，特别是景区周边，提早出台居民安置办法。优先发展公共交通。

　　强化城市管理。加强市容环境建设，完成国家卫生城市年度创建任务。健全长效机制，实施规范化、常态化、精细化管理。做好延西高速铜川新区出入口景观建设、待建地段美化、主干道街景、标志性建筑亮化。规范路名、街名和标志标牌设置。抓好城市出入口、城乡结合部和背街小巷的环境整治，做到垃圾日产日清，全天保洁。规范住宅小区物业管理。

　　（四）加快城镇化建设，推进城乡一体化发展

　　抓住铜川被列入全省城乡统筹重点示范区的机遇，科学规划，因地制宜，促进城乡共同繁荣，逐步实现城乡一体和公共服务均等化。

　　发展壮大县域经济。赋予区县更大的自主权。完善县域经济发展考核机制，强化对标管理，促进区县在竞争中争先进位。立足实际，突出区域特色，着力抓好大企业带动、产业集群培育、园区建设、现代农业、物流等重点工作，增强县域经济综合实力和统筹城乡发展能力。

　　加快重点镇建设。继续支持宜君秀美山城建设。加快黄堡示范镇、照金红色旅游名镇和陈炉历史文化名镇建设，统筹做好建制镇建设，增强城镇产业发展、公共服务、吸纳就业、人口聚集功能。推动农民居住向城镇集中、工业企业向工业园区集中、耕地向规模经营集中，向二、三产业转移就业，实现1.4万农村居民进城落户。

　　改善农村生产生活条件。政策一致、规划一体、服务均等，以区县为单位，整合项目和资金，推进7个新型农村社区建设，统筹解决好搬迁农民生产生活、就业社保、教育医疗、文化体育等问题，坚决避免复制农村。建成县乡、通村公路100公里。解决2万农村人口饮水安全问题。农村危旧房改造3000户。加强村庄环境整治，落实长效化、专业化管理措施，持续改善农村环境状况。

　　（五）加强生态文明建设，努力建设美丽铜川

　　建设生态文明，是关系人民福祉、关乎未来发展的长远大计。顺应人民群众对美好生活环境的期待，切实加强生态建设和环境保护，让铜川的天更蓝、地更绿、水更清！

　　推进自然生态系统建设。市区县齐抓共管，继续做好天保公益林、退耕还林、三北防护林等林业重点工程建设，大力实施城市绿化、道路绿化、景区绿化、流域绿化和家园绿化五大工程。巩固省级园林城市创建成果，加快药王生态湖建设，新增城市绿地60万平方米，人均达到11.4平方米。提高包茂高速、210国道铜川段绿色长廊建设和管理水平，抓好耀旬路景观系统建设。开工建设铜川植物园。全年植树造林16万亩，治理水土流失面积150平方公里。实施漆水河黄堡段、印台段和沮河锦阳段及清河彭镇段综合治理工程，提高河道设防标准。

　　加大环境保护力度。推进省级环保模范城市创建工作，完成华能铜川电厂2号机组及4户水泥生产线脱硝工程，开展PM2.5数据监测和发布工作。完成药王山景区第一批7户污染企业搬迁，集中整治小石碴厂、小石灰窑、小矿山的环境污染。深化市区煤烟污染治理，新增天然气用户1万户，城市空气质量二级以上天数超过325天。加快北市区污水处理厂中水回用、新耀污水处理厂污泥处置工程建设，建成北市区生活垃圾处理厂和徐家沟、鸭口煤矿生活污水处理工程，确保出市断面水质达到功能区要求。加强农村面源污染治理，防止污染向农村转移。

　　节约集约利用资源。力争将我市列入全国节能减排试点城市。强化新设备、新技术、新工艺的运用，抓好水泥低温余热发电工程，全面推进工业、建筑、交通和公共机构的节能。万元工业增加值用水量下降1%。实行用地增减挂钩，开发整理土地6400亩，基本农田保护面积111万亩。完成11个矿山地质环境治理项目。

　　（六）深入推进改革开放，增强经济发展活力

　　改革开放是加快发展的动力源泉。用改革的红利释放发展潜力，用开放的思路借助外力发展，用人才的智慧提升核心竞争力，最大限度地增强经济发展活力。

　　深化重点领域改革。争取中省更多的政策试点。加快铜变公司、耀县水泥厂等市属国有企业改革，推进国有企业厂办大集体改革。加强国资监管，盘活置换土地、厂房等有效资产。做好统筹科技资源改革工作。实现区县级公务卡改革全覆盖，将非税收入全部纳入收缴系统。市政府组成部门的预算全部公开。引进长安银行在我市设立分行，推进农村信用社改革和村镇银行、小额贷款公司、担保公司等地方性金融机构建设。强化价格监测调控，推进价格市场化改革。加大价格调节基金征收力度。深化文化体制改革。抓好县级公立医院改革。以推行居住证为重点深化户籍制度改革。推进事业单位分类改革，加强对行政事业单位经营性国有资产的管理。

　　大力发展非公经济。认真落实鼓励支持非公经济发展的政策措施，营造公平公正竞争的发展环境。通过放宽市场准入、政府资金引导、给予政策优惠等手段，大力扶持创新型、成长性好的非公企业。新增私营企业300户，小微企业和经营户3000户。加快信用担保体系建设，引进培育新兴融资组织机构，着力解决中小企业融资难题。建立非公经济发展激励机制，对在吸纳就业、名牌创建等方面做出贡献的企业给予奖励。非公经济增加值占生产总值的比重达到48%以上。

　　着力培育外贸增长点。加快出口示范基地建设，大力培育外贸骨干企业，扩大鲜苹果及深加工、中医药及植物提取物、服装等地产品的出口。全年实现外贸进出口总额1400万美元。

　　激发人才创造活力。敞开大门，以政策引人，以环境赢人，以事业留人，让铜川成为有本领、有作为、有理想的各类人才向往的地方。支持企业技术创新，加快科技成果转化和推广应用，推动区域经济创新发展。

　　（七）着力保障和改善民生，让发展成果惠及更多群众

　　坚持将新增财力和财政支出的80%用于改善民生，带着责任，带着感情做好民生工作。

　　办好人民满意的教育。坚持教育优先发展。均衡发展义务教育，合理配置教育资源，着力解决部分区域学校大班额问题。支持新区科学布局中小学校，加快耀州区“双高双普”创建步伐，启动宜君县“双高双普”创建工作。新建公办幼儿园19所。大力发展职业教育。支持特殊教育。对400名新录取家庭经济困难大学生实施资助，对6000名家庭经济困难大学生实施生源地助学贷款。保障农民工子女平等接受义务教育。

　　努力扩大就业。完善就业服务体系，引导劳动者转变就业观念，鼓励多渠道多形式就业。加强职业技能培训，提升劳动者就业创业能力。以小额担保贷款和孵化基地建设为抓手，促进创业带动就业。全年发放小额担保贷款2.2亿元，新增就业人员1.6万人。实施收入倍增计划，深化企业和机关事业单位工资制度改革，落实新的企业最低工资标准，多渠道增加群众经营性、工资性和财产性收入。

　　加快保障房建设。实行工程质量终身负责制。新开工保障房7600套，解决1万户困难家庭住房问题，新增租赁补贴户1500户。加快锦绣新城建设。加快地质灾害隐患点群众避险搬迁，力争完成总体搬迁任务的70%以上。

　　完善社保服务体系。全市企业职工基本养老保险做到应保尽保，城乡居民社会养老保险参保29.3万人，参保率98%。年内实现医疗保险省内异地结算，全市城镇基本医疗保险参保37万人，参保率95%。将城乡居民医保补助标准提高到每人每年300元。在全市乡镇卫生院推行“先住院、后结算”服务。打造铜川养老服务品牌，建设市养老服务中心、宜君县养老服务中心和耀州邻德社区、印台新建社区、陈炉镇老年日间照料中心。加快20个城市社区服务体系项目、14个农村社区服务中心建设。建成耀州区、王益区、印台区残疾人康复中心和托养中心，推进残疾人辅助器具适配工作，确保3年内实现全覆盖。

　　提高健康服务水平。大力开展群众性爱国卫生运动，加快健康城市信息化建设，健全农村三级医疗卫生服务网络和城市社区卫生服务体系，完善乡镇卫生院规范化管理，推进乡村卫生服务一体化。提高县、乡、村和社区卫生服务机构国家基本药物使用率。实施妇女“两癌”筛查和救助工程、“母亲健康工程”和“优生促进工程”，提高出生人口素质，促进人口均衡发展。

　　丰富群众精神文化生活。举办陕甘边革命根据地创建80周年纪念活动。加快文化活动场馆建设。完成2万套广播电视“户户通”设备安装和5万户城市有线电视双向网改造任务。加强文化遗产和文物保护。建立政府购买公共文化服务机制，深入开展“唱响铜川”等群众文化活动。实施体育惠民工程，逐步开放体育场馆，完成20个行政村农民体育健身工程和5个社区体育器材配送工程，促进群众体育和竞技体育全面发展。

　　（八）加强和创新社会管理，努力构建和谐社会

　　以人为本，服务为先，夯实管理基础，创新服务模式，让群众享受和谐生活。

　　确保安全生产。加强以落实基层责任、强化基础能力、提升基本素质为内容的安全生产“三基”建设，全面落实各级政府安全生产组织领导责任、部门行业监管责任和企业主体责任。严格落实企业法定代表人安全生产责任制，强化基层安全生产责任，健全隐患排查治理常态机制。深入开展打非治违、隐患排查治理、安全生产标准化建设和安全社区建设。以事故超前预防为主线，突出抓好煤矿、道路交通、非煤矿山和建筑施工等行业领域的安全专项整治，强化过程控制和精细化管理，努力降低事故总量、遏制较大事故、杜绝重特大事故，促进安全生产形势持续稳定好转。加强食品药品安全监管，实施放心粮油全覆盖工程，严厉打击食品药品领域的违法犯罪行为，确保群众饮食用药安全。

　　推进平安建设。深入开展“平安铜川”创建活动，加强社会治安综合治理，着力建设立体化防控体系，提高社会治安防控能力，让群众享受平安。抓好学校安全及周边环境综合治理工作。加强应急管理，及时发布预警信息，全面落实各项应急预案，提升应对突发事件能力。

　　加强基层基础建设。加强社会管理和服务体系建设，建成四级便民服务网络。巩固“双拥”共建成果，加强国防动员和后备力量建设。支持工会、共青团、妇联、科协等人民团体更好地发挥联系和服务群众的作用。做好农民工、流动人口、空巢老人、留守儿童等特殊群体的服务工作。加强对网络等媒体的管理。深入推进“六五”普法依法治理。做好民族、宗教工作。强化诚信教育，建设覆盖全社会的诚信系统。完善矛盾纠纷排查化解机制，将矛盾纠纷化解在基层和萌芽状态，确保实现“一控制、两下降”目标。

　　三、加快服务型政府建设

　　打铁还需自身硬。深入解放思想，有精神、在状态，始终勤勉敬业，在服务中实施管理，在管理中实现服务，建设为民、务实、清廉的服务型政府。

　　全面推进依法行政。转变政府职能是深化行政体制改革的核心。坚持市场化改革方向，推进政企分开、政资分开、政事分开、政社分开，做到不越位、不缺位、不错位。依法行政是政府工作的准则。严格按照法定权限和程序行使权力，依法依规做事，讲规矩、讲程序，自觉接受人大及其常委会的法律监督和工作监督，主动接受人民政协的民主监督，认真听取民主党派、工商联、无党派人士和各人民团体的意见。健全重大行政决策事项听取人大、政协意见制度，推行重大行政决策草案社会公布制度，完善听证会、网上征询等公众参与决策机制。

　　加大政务公开力度。创新政府服务管理方式，充分利用网络平台，扩大政务公开，保障群众的知情权、参与权、表达权和监督权。扩大部门预算、“三公”经费、专项资金的公开范围，探索政府投资项目竣工决算的审计结果公开。重视舆情研判、引导和处置，及时办理群众来信和网民留言，虚心听取并认真回应民声民意，妥善协调处理各方面的利益诉求。加强政府门户网站建设，完善政务微博等公开渠道。

　　切实改进工作作风。坚决贯彻中央“八项规定”和省、市委的实施意见，深入开展“为民服务零距离”活动。精简会议活动，改进会风文风。整治“庸懒散”，激活精气神，释放正能量。强势推进抓落实。最好的落实就是一抓到底，最大的公信就是说到做到，大力倡导低调务实、少说多干、干就干好。向社会公布行政职权及运行流程，严格实行首接首办制，坚决纠正吃拿卡要、推诿扯皮、效率低下等行为。完善目标责任考核，加强效能监察，开展政府部门履职评估，完善落实行政问责制，盯住不落实的事，追究不落实的人，切实提高政府的公信力和执行力。

　　着力加强廉政建设。全体政府工作人员必须严格遵守廉政准则，加强人格修为，严守纪律和道德底线。认真落实廉政建设责任制，加强廉政风险防控管理。深入开展纠风和专项治理，坚决纠正教育、医疗、涉农、征地拆迁、保障房、环境保护等领域的不正之风。规范公务消费，厉行勤俭节约，反对奢侈浪费。加大查办案件工作力度，切实做到干部清正、政府清廉、政治清明。

　　各位代表，富民强市的目标引领着我们，提速转型发展的任务激励着我们，美好的未来召唤着我们。让我们以党的十八大精神为指引，在市委的坚强领导下，解放思想，真抓实干，为建设经济强、文化兴、生态美的全国知名休闲养生城市而努力奋斗!
